# *De Novo* Transcriptome Assembly from Fat Body and Flight Muscles Transcripts to Identify Morph-Specific Gene Expression Profiles in *Gryllus firmus*

**DOI:** 10.1371/journal.pone.0082129

**Published:** 2014-01-08

**Authors:** Neetha Nanoth Vellichirammal, Anthony J. Zera, Rudolf J. Schilder, Cody Wehrkamp, Jean-Jack M. Riethoven, Jennifer A. Brisson

**Affiliations:** 1 School of Biological Sciences, University of Nebraska-Lincoln, Lincoln, Nebraska, United States of America; 2 Bioinformatics Core Research Facility, University of Nebraska-Lincoln, Lincoln, Nebraska, United States of America; 3 Department of Biology, Penn State University, State College, Pennsylvania, United States of America; Oxford Brookes University, United Kingdom

## Abstract

Wing polymorphism is a powerful model for examining many aspects of adaptation. The wing dimorphic cricket species, *Gryllus firmus*, consists of a long-winged morph with functional flight muscles that is capable of flight, and two flightless morphs. One (obligately) flightless morph emerges as an adult with vestigial wings and vestigial flight muscles. The other (plastic) flightless morph emerges with fully-developed wings but later in adulthood histolyzes its flight muscles. Importantly both flightless morphs have substantially increased reproductive output relative to the flight-capable morph. Much is known about the physiological and biochemical differences between the morphs with respect to adaptations for flight versus reproduction. In contrast, little is known about the molecular genetic basis of these morph-specific adaptations. To address this issue, we assembled a *de novo* transcriptome of *G. firmus* using 141.5 million Illumina reads generated from flight muscles and fat body, two organs that play key roles in flight and reproduction. We used the resulting 34,411 transcripts as a reference transcriptome for differential gene expression analyses. A comparison of gene expression profiles from functional flight muscles in the flight-capable morph versus histolyzed flight muscles in the plastic flight incapable morph identified a suite of genes involved in respiration that were highly expressed in pink (functional) flight muscles and genes involved in proteolysis highly expressed in the white (histolyzed) flight muscles. A comparison of fat body transcripts from the obligately flightless versus the flight-capable morphs revealed differential expression of genes involved in triglyceride biosynthesis, lipid transport, immune function and reproduction. These data provide a valuable resource for future molecular genetics research in this and related species and provide insight on the role of gene expression in morph-specific adaptations for flight versus reproduction.

## Introduction

Wing polymorphism is a phylogenetically-widespread and ecologically-important feature of insects [1]–[4]. The polymorphism consists of a flight-capable phenotype (morph) that has fully developed wings and flight muscles, and one or more flightless morphs that have reduced wings and/or flight muscles. Importantly, loss of flight ability is correlated with substantially increased egg production, especially during early adulthood [1], [2], [4], [5]. Thus, wing polymorphism is a classic example of an adaptive trade-off in which enhancement of one function (e.g. egg production) has evolved at the expense of another function (e.g. flight ability) [1], [2], [6]–[8]. The polymorphism occurs commonly in many insect groups such as aphids, beetles, crickets, grasshoppers, and true bugs where it often plays a key role in adaptation to temporally or spatially varying demands for dispersal versus reproduction [2], [5]. Since the mid-1900s, wing polymorphism has been used extensively as an experimental model to investigate a number of important topics in evolution, most notably, the evolution of dispersal and flightlessness [1], [4], [9], development [7], [8], [10], physiology [6], [8], [10], and life histories [5], [6].

Despite intensive investigation, a number of important aspects of wing polymorphism remain understudied, such as global variation in gene expression that underlies morph adaptations. Thus far, such studies have been reported only for two wing-polymorphic species of aphids [11], [12]. Obtaining data on morph-specific transcriptome abundance for physiologically well-studied species would provide key information on the molecular mechanisms underlying physiological differences between morphs, in addition to providing a wealth of gene sequences that could be used for future molecular studies of adaptation. The advent of cost-effective next-generation sequencing has opened up new avenues for global, whole-genome analyses of gene expression in ecologically interesting non-model organisms [13]–[15]. It is now possible to sequence and assemble a *de novo* transcriptome and use it to perform genome-wide gene expression profiling for these species without reference genome sequence information [16], [17].

Here we report on the construction of the *de novo* transcriptome of the wing polymorphic cricket, *Gryllus firmus*, a species that is especially well-studied with regard to morph-specific physiological-genetic aspects of development and life history evolution (Table 1). We use this *de novo* transcriptome to compare morph-specific differences in transcript abundance in the context of adaptations for flight versus reproduction. As is typical for wing-polymorphic species, *G. firmus* produces a flight-capable morph that has fully-developed and functional wings and flight muscles during adulthood (designated LW(f) = longed winged with functional flight muscles) and which delays egg production [3], [6], [8]. The species also has two types of flightless morphs that differ in the timing of attainment of flightlessness, but each of which exhibits four-fold greater fecundity than the flight-capable morph by the end of the first week of adulthood. One flightless morph emerges as an adult with shortened wings and vestigial, non-functional flight muscles (designated as SW), and is obligately flightless throughout adulthood [18]. A second flightless morph initially has fully-developed wings, functional flight muscles and can fly. During mid-adulthood this plastic, flightless morph (designated as LW(h) = long-winged with histolyzed muscles) degenerates its flight muscles, loses the ability to fly, and increases egg production to levels seen in the obligately flightless morph [18]. Thus, in the laboratory or field, populations can consist of LW(f), LW(h) or SW morphs of the same age.

**Table 1:** Differences in morphological, reproductive, physiological, biochemical and molecular traits among adult morphs of *Gryllus firmus*.

Trait	Morph difference	
Wing length	LW(f) = LW(h)>SW	[4], [8], [18]
Flight muscle mass	LW(f)>LW(h)>SW	[4], [8], [18]
Ovarian growth and egg production	SW = LW(h)>LW(f)	[4], [8], [18]
Respiration rate	LW(f)>SW	[4], [8], [18], [19]
Specific activities of glycolytic and lipid-oxidizing enzymes in flight muscles	LW(f)>LW(h)>SW	[20]
Lipid reserves accumulated during adulthood	LW(f)>SW	[8], [21], [22]
Rate of total lipid and triglyceride biosynthesis	LW(f)>SW	[8], [22], [23]
Relative rate of triglyceride vs. phospholipid biosynthesis	LW(f)>SW	[8], [21], [22]
Rate of fatty acid and amino acid oxidation	SW>LW(f)	[8], [22], [24], [25]
Conversion of amino acids into ovarian protein	SW>LW(f)	[25]
Conversion of amino acids into lipid	LW(f)>SW	[25]
Specific activities of lipogenic enzymes	LW(f)>LW(h) = SW	[22], [26]
Transcript abundance of lipogenic enzymes	LW(f)>SW	[27], [28]
Concentration of lipogenic enzymes	LW(f)>SW	[27], [28]
Kinetic properties of purified lipogenic enzymes	LW(f) = SW	[27], [28]

LW(f) = longed-winged morph with functional flight muscles, LW(h) = long-winged morph with histolyzed muscles, SW = short-winged morph with non-functional flight muscles.

As is typical for wing-polymorphic orthopterans, variation in wing morph in *Gryllus* is under polygenic control and is also influenced by a variety of environmental factors such as density, photoperiod and nutrition. Sampled populations near Gainesville, Florida, contain LW(f), LW(h) and SW morphs during the same time period [8]. Most previous studies of the physiological underpinnings of wing-polymorphism in *Gryllus* have been conducted on genetic stocks artificially-selected for wing morph, and raised under constant environmental conditions (see Table 1), and thus have focused on the physiological-genetics of wing polymorphism. These same stocks have been used in the transcriptome studies described below.

In the present study we focused on morph-specific differences in transcript abundance in flight muscle and fat body of *G. firmus*, two key organs involved in morph specialization for flight or egg production. We sequenced and assembled a *de novo* reference transcriptome using mRNA collected from these two organs. We then identified differentially expressed transcripts between the functional and histolyzed flight muscles of the LW(f) and LW(h) morphs, respectively, to discover genes involved in the degeneration of flight muscles of the plastic, flightless morph, as well as functional differences between flight muscles of these two morphs important in the flight/fecundity trade-off. For example, previous studies demonstrated that reduced respiration of histolyzed flight muscle appears to be an important factor which frees nutrients for the increased ovarian growth of the LW(h) morph [18]. Yet, little is known about the biochemical or molecular basis of either muscle histolysis or reduced respiration. Flight muscles of the SW morph were not characterized, because, during the molt to adulthood, the flight muscles are already substantially reduced, sometimes only consisting of the sheath surrounding the muscle. We also compared the transcriptome profiles between fat body of the flight-capable LW(f) and flight-incapable SW morphs and identified genes that were differentially expressed. Fat body is the major organ of intermediary metabolism, and has been extensively studied in regard to differences in lipid and protein metabolism between adult LW(f) and SW morphs important for production of lipid flight fuel or ovarian protein by focusing on key candidate proteins and pathways, but no previous transcriptome-wide analysis has been performed (Table 1).

## Materials and Methods

### Insect stocks

*Gryllus firmus* used in the present study were females taken from artificially selected, outbred populations that are nearly pure-breeding for the long-winged (LW) or short-winged (SW) morph (Block-2 lines; see [22] for details of artificial selection). These stocks were initiated using field-collected LW and SW *G. firmus* and were the same stocks used in previous studies of lipid metabolism and juvenile hormone endocrinology [3], [22], [23]. Populations were maintained under standard conditions as described previously (e.g., 28°C, 16L: 8D photoperiod; see [22] for details).

### Sample collection

Fat body samples were obtained either early in the 16 h photophase (three to four hours after lights on) or late in the photophase (within two hours prior to lights off) from day-5 LW(f) or SW female *G. firmus* (day 0 = day of molt to adulthood). Day 5 of adulthood is the age of physiological and biochemical comparisons undertaken between these morphs previously [22]. On this day, morphs differ substantially in ovarian growth and aspects of intermediary metabolism related to morph-specializations for flight (e.g. enhanced lipid biosynthesis in the LW(f) morph) and reproduction (enhanced ovarian growth in the SW morph). Organs were sampled during two times of the photophase to determine if morph differences are contingent upon time-of-day, as is the case for the hemolymph juvenile hormone level [29]. Within and between morph comparisons of data from different times of the day are the subject of a future study; the present study focuses on differences that were consistently different between morphs regardless of the time of day. Pieces of fat body were removed from individual female crickets with forceps and were placed on a piece of parafilm on ice until sufficient amount of material was obtained (about three minutes). Tissue from each individual cricket was then dropped into a 1.5 mL Eppendorf tube and flash frozen with liquid nitrogen. Tubes were capped after the nitrogen had evaporated and were stored at −86°C until RNA extraction.

All flight muscles were removed early in the photophase. Pink (functional) or white (histolyzed) thoracic (dorsolongitutinal and dorsoventral) flight muscle was removed from day 5 LW(f) or LW(h) female crickets. By the end of the fifth day of adulthood, about 80% of adult females have retained their pink, functional flight muscles. In the other 20% of females, muscles have histolyzed, becoming slightly smaller and white in color [30]. The proportion of LW individuals with histolyzed flight muscles increases with the age of the cricket [18]. LW(f) crickets are capable of flight at about day 5 of adulthood. Collected muscle tissue was dropped directly into a 1.5 mL Eppendorf tube and flash frozen with liquid nitrogen. Tubes were capped after the nitrogen had evaporated and were stored at −86°C until RNA extraction.

### cDNA library preparation and sequencing

cDNA libraries were separately constructed from the pink flight muscles of the LW(f) morph, white muscles of the LW(h) morph, and fat body of SW and LW(f) morphs using the following protocol. Poly(A) RNA was extracted directly from the tissues with the Micro-Poly(A)-Purist Kit (Ambion). mRNA was fragmented with fragmentation reagents (Ambion) at 70°C for 3 min 50 sec, column purified (RNA Clean & Concentrate kit-5, Zymo Research) and then subjected to first strand cDNA synthesis using oligo-dT, random hexamer primers and Superscript II reverse transcriptase (Life Technologies, Inc). Second strand cDNA was synthesized using RNaseH and DNA polymerse I (Epicentre). These cDNA fragments were column purified (DNA Clean & Concentrate Kit-5, Zymo Research) end-repaired (End-It DNA End Repair kit, Epicentre) and A-tailed using Klenow enzyme (NEB). An index adapter was ligated to the cDNA using Fast-Link DNA ligation kit (Epicentre). The library was size selected for 200–250 bp, enriched by PCR (Phusion DNA polymerase, Finnzymes), and purified using a PCR purification kit (Zymo Research) to create the final cDNA library. Tagged libraries were pooled in equal amounts and sequenced on an Illumina Genome analyzer II flow cell. Three biological replicates per tissue type (3×LW(f) flight muscle, 3×LW(h) flight muscle, 3×LW(f) fat body early photophase, 3×LW(f) fat body late photophase, 3×SW fat body early photophase, 3×SW fat body late photophase) were sequenced in a total of five lanes. An additional four fat body samples, two from LW(f) and two from SW, were initially sequenced in an additional lane for a feasibility study. These four sequences contributed to the *de novo* transcriptome assembly but were not used for differential gene expression analysis. The raw sequence reads for *G. fimus* are deposited in NCBI's Short Read Archive (SRA) under BioProject id PRJNA198853, with sample identifiers SAMN02058407 to SAMN02058428.

### *De novo* transcriptome assembly

For the transcriptome assembly, we performed both guided and unguided assemblies in order to identify the best assembly. The default Illumina quality filter pipeline (CHASTITY> = 0.6) was used to identify sequences with low signal to noise ratio. These were removed from further analysis. Sequences from both tissue types (muscles and fat body) were pooled for the assembly. For the guided assembly, *G. firmus* sequence reads were aligned via Bowtie2 (version 2.0.0-beta6; [31]) to 32,159 mRNA's from *G. bimaculatus* downloaded from NCBI nucleotide database. Bowtie2 was run with default parameters (end-to-end sensitive matching, report best hit). Matching reads were extracted via *samtools* (version 0.1.18 [32]) using parameter setting –F 12 (skip unmapped reads) and converted into regions via *bamToBed* in the bedtools software package (version 2.16.1 [33]), and FASTA sequences of these regions were extracted via *fastaFromBed* (bedtools) from the mRNAs to serve as the conserved reference regions for the transcriptome assembly. Velvet (version 1.2.07) followed by Oases (version 0.2.08) was used for both the *de novo* and guided transcriptome assemblies with a k-mer value of 57 [34], [35]. We tried a range of k-mer values from 13 to 57 and settled on 57 since it gave us, on average, longer contigs (data not shown). Velvet is specifically for genomic assembly, however it was a necessary first step since Oases takes the preliminary assembly results from Velvet and clusters the contigs into mRNA loci and, where possible, also delineates isoforms. For the initial Velvet step we used automatic determination of expected coverage and cutoffs (-exp_cov auto) as these were very similar to manual cutoffs determined by visual inspection (expected coverage 10.3, cutoff 5.1). Specifically for the guided assembly, we aligned all *G. firmus* reads via Bowtie2 (with default parameters and –M 10, allowing up to 11 hits) against the extracted conserved reference sequence from *G. bimaculatus*, and produced a SAM file that together with the reference sequence was input to the Columbus module of Velvet to produce the assembly. The minimum reported contig length (--min_contig_lgth) for both assemblies was set to 100 bases.

Oases determined the transcripts for both the *de novo* and the guided assembly, and if there were multiple transcripts per locus, a representative transcript was chosen using the highest Oases transcript scoring confidence and the longest length using an in-house Perl script. Based on a lower N50 in the guided assembly (302 versus *de novo* 513 nt) and a poor nucleotide similarity between the guided *G. firmus* transcript assembly and *G. bimaculatus* mRNA (67%), the *de novo* transcriptome assembly with 34,411 transcripts was selected for downstream analysis.

To gain insight in the completeness of the *de novo* transcriptome, we performed two analyses. First, we randomly selected from 10% to 100% of the total number of reads and assembled the *de novo* transcriptome at each 10% increment. Oases reports the number of mRNA transcripts and loci, with multiple transcripts (isoforms) per loci possible. As a simple measure of completeness as to the total genes discovered, the number of mRNA loci should start to plateau with increasing reads.

The second analysis estimated the proportion of completeness of each transcript that was assembled via the ortholog hit ratio (OHR, [36]), where an OHR near to 1.0 would indicate a relatively complete assembly of that transcript. We compared the assembled transcripts to 620 *G. firmus* EST's previously sequenced from the accessory gland, and to 32,159 mRNA transcripts from *G. bimaculatus* (Table S1 and S2); both data sets were downloaded from Genbank in May 2012. Based on the best hit for each transcript, the OHR was calculated on the number of perfect matching bases in the alignment divided by the length of the EST.

This Transcriptome Shotgun Assembly project has been deposited at GenBank under the accession GAIZ00000000.

### Functional annotation and downstream processing of the assembled transcriptome

The *de novo* assembled transcripts of *G. firmus* were annotated by performing a BLAST search with the software Blast2GO v.2.6.0 [37]. The BLASTx search was done with cut-off E-values of 1.0e^−1^ and 1.0e^−3^ against the non-redundant protein sequence database. The same software was used to obtain the gene ontology (GO) information for the assembled transcripts describing different biological processes, molecular function and cellular components [15]. Default parameters *i.e.*, the best 20 BLAST hits with a cut-off E-value of 1.0e^−6^ and annotation cut-off of 55%, were used for the GO annotation.

### Differential gene expression profiling for flight muscles and fat body in *G. firmus*

For differential gene expression analysis, the sequences obtained from each sample were mapped against the *de novo* assembled transcriptome using the software Arraystar v.5 of the DNASTAR Lasergene core suite using the following parameters. Briefly, reads were aligned to the reference transcriptome if they had 80% of the bases matching within each read, with no more than three mismatches. Reads mapping to more than 15 positions were excluded from the analysis. RPKM (reads per million kilobases per million mapped reads) was used to normalize the read counts between the samples. Genes with low read counts (less than an average of 10 total counts in all samples within an experiment, i.e., muscles or fat body) were excluded from the analysis in order to focus on transcripts with good quality expression evidence. Differentially expressed (DE) genes were identified using t-tests with FDR correction of significance values [38]. The fold change of each DE gene was calculated by comparing the averaged RPKM across replicates. A gene was considered significantly differentially expressed if its corresponding FDR corrected *p*-value was ≤0.05 and if its fold change was ≥1.5. The transcriptomes of white and pink muscles from the LW morph were compared to identify genes that differed in their expression between the two muscle types. Gene expression profiles of fat body from LW(f) and SW morphs collected in the morning and evening were pooled within each morph and compared to identify genes that differed in their expression between the two morphs. To examine global differences in fat body between LW(f) and SW morphs, we identified genes with FDR corrected *p*-values≤0.05 and fold changes ≥1.5. When we examined candidate genes and associated pathways for fatty acid and triglyceride biosynthesis, we did not filter our significant gene list using these same criteria. Rather, we included some genes with uncorrected *p*-values<0.05 and fold changes <1.5 because we had *a priori* expectations that these specific genes were differentially expressed [22], [27], [28] and therefore false discovery rate corrections based on the total number of expressed genes were not required. To identify pathways associated with DE genes, enrichment analysis for GO pathways using Blast2GO v.2.6.0 was performed.

### Validation of differentially regulated genes by qRT-PCR

Quantitative reverse-transcriptase polymerase chain reaction (qRT-PCR) for differentially expressed genes in the fat body of LW and SW morphs identified from RNA-seq data was performed for validation. An additional four biological replicates of fat body samples were collected from day 5 LW(f) and SW morphs using the same procedure as mentioned before. RNA was isolated using the RNeasy Mini kit (Qiagen), DNase (Ambion) treated and converted to cDNA using random hexamer primers (Invitrogen) and Superscript II reverse transcriptase (Life Technologies, Inc). Primers for seven genes were designed using Primer3Plus [39] (Table S3). qRT-PCR reactions were performed on a Real-Time PCR system 7500 (Applied Biosystems) using SYBR green PCR master mix (Applied Biosystems). We tested three genes (actin, vesicle trafficking protein and calcium binding protein) for stable expression profiles across all samples using Normfinder V2 [40], and selected actin as the best reference gene. Each biological replicate was measured with two technical replicates and fold change was calculated using the 2^−ΔΔ*C*T^ method [41]. The correlation between the expression values obtained by RNA-seq and qRT-PCR was calculated using Spearman's correlation coefficient.

## Results and Discussion

### RNA sequencing, *de novo* assembly and validation of *G. firmus* transcriptome

Sequencing of *G. firmus* transcripts yielded 141.5 million, 76-mer single end reads from a total of 22 samples and two tissue types (44 million reads from muscles and 97.5 million reads from fat body). The average reads obtained per sample was seven million. We performed both guided and unguided assemblies of the transcripts with the goal of producing a reference transcriptome. The guided assembly produced a larger draft transcriptome, but with a lower N50, the contig length for which half of all bases in the assembled sequences are in a sequence equal or longer than this contig length (Table 2). After transcript determination by Oases, the guided and *de novo* assemblies had 37,707 and 37,391 transcripts respectively. Both assemblies were further processed to select only one representative transcript per locus based on the highest Oases confidence score, and the longest length, in that order (in-house Perl script, available upon request) after which the two assemblies did not differ much in length and number of representative transcripts (34,668 versus 34,411 for the guided and *de novo*, respectively). Since N50, an important quality measure for assemblies, is lower in the guided assembly (302 versus 513 nt) and the nucleotide similarity between the guided *G. firmus* transcript assembly and *G. bimaculatus* mRNA was only 67% (and thus sequence from *G. bimaculatus* is not optimum to serve as a guide), the *de novo* transcriptome assembly with 34,411 transcripts was selected for downstream analysis. The minimum length of the transcripts is 100 nt, with more than 75% of the transcripts having a length of less than 500 nt (see Figure S1).

**Table 2:** *De novo* and guided transcriptome assembly details.

		*De novo*	Guided
**Velvet**	Number of contigs	33,849	57,665
	N50 (median length, nt)	513	302
	Total length of all contigs (nt)	11,471,107	15,770,700
**Oases** *	Number of loci	34,411	34,668
	Number of transcripts	37,391	37,707
	Total length (nt)	18,832,621	18,889,750
**Representative**	Number of transcripts	34,411	34,668
	Total length (nt)	13,042,341	13,096,374

Oases clusters the initial assembly from Velvet into gene loci and determines the existence, if any, of isoforms. The representative transcripts per loci were extracted via an in-house Perl script (available upon request), using both the confidence and length of the transcripts as the determining factors.

Two analyses showed that our sequencing captured a subset, but not the entirety, of the *G. firmus* expressed genome. First, by assembling the *de novo* transcriptome with an increasing proportion of the total sequence reads, we found that the number of transcripts did not plateau (Table S4). A plateau would have indicated completeness. And second, we calculated ortholog hit ratios (OHRs) for our *G. firmus* transcripts compared to 620 *G. firmus* and 32,159 *G. bimaculatus* sequences downloaded from Genbank. Figure S2 shows OHRs in terms of assembly coverage and length of the assembled transcripts. The median OHR for the transcripts matching *G. firmus* ESTs was 0.23, while the median OHR for those matching the *G. bimaculatus* mRNAs was 0.94. The lower median OHR for the former is likely due to the low number (620) of *G. firmus* ESTs in Genbank and due to the fact that these ESTs are primarily from accessory gland, not from fat body or muscles.

We conclude from these analyses that deeper sequencing is required to obtain a more complete transcriptome. We did not expect to capture the full extent of *G. firmus* transcripts given that we sampled from only two tissue types, fat body and flight muscles, rather than from a suite of tissues and developmental stages that would be necessary to produce a fully representative transcriptome. Our purpose here was not to produce a definitive transcriptome for this species. Rather, our purpose was to identify expressed transcripts in these two tissue types and compare them between cricket morphs.

### Functional annotation of the *G. firmus* transcriptome

The *de novo* transcriptome of *G. firmus* was compared against the non-redundant protein sequence database using the software Blast2GO. Out of the 34,411 transcripts, 14095 (41.5%) had a BLAST hit with an E-value cutoff of 1.0e^−3^. Decreasing the stringency of BLASTx search cut-off E-value to 1.0e^−1^ only slightly increased the number of BLAST hits (42.1%) and these BLAST results were used for annotating the transcripts. The majority of the sequences with BLAST results had top hits to genes from insect species as expected, especially *Tribolium castaneum*, *Pediculus humanus*, and *Nasonia vitripennis* (Figure S3). 12,427 transcripts (36%) were linked to GO annotations via Blast2GO. 3823 transcripts coded for enzymes with unique Enzyme Commission (EC) numbers.

**Figure 1:**
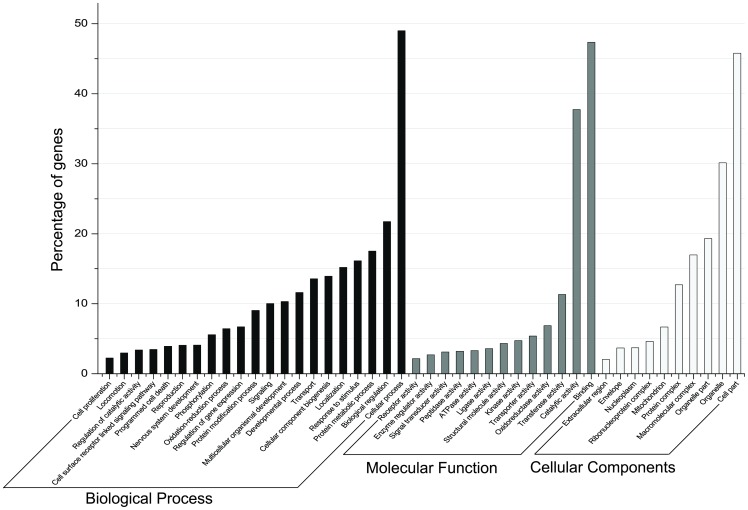
Gene ontology classifications of the assembled *G. firmus de novo* transcriptome. Bars show the percentage of *G. firmus* transcripts annotated for biological process, cellular component and molecular function GO categories.

The GO term distribution of different biological processes, molecular functions, and cellular components of annotated genes of *G. firmus* is shown in Figure 1. The overall distribution of GO classifications was very similar to other *de novo* insect transcriptomes (e.g., [42]). Among GO terms associated with biological processes, genes involved in cellular process, biological regulation, protein metabolic process, response to stimulus and localization were abundant. Genes involved in binding (ATP, protein, RNA and DNA binding) and catalytic activity (oxidoreductase, hydrolase, peptidase, kinase, ATPase, and ligase activity) were highly represented in the molecular function category. These categories indicate a high state of metabolic activity and regulation of gene expression in *G. firmus* muscle and fat body.

### Fat body and muscles express different subsets of the *de novo* transcriptome

Reads generated from each sample were mapped to the *de novo* transcriptome with Arraystar v.5 (DNAStar). Average mapping percentages were 64% and 54% for flight muscles and fat body, respectively. The lower mapping efficiencies in both the tissues are due to the removal of singletons, low coverage nodes during assembly, and transcripts of length smaller than 100 nt. Even though the RNA isolation protocol included steps to remove ribosomal RNA and enrich mRNA, it was less effective in flight muscles. 16SrRNA reads were abundant in flight muscle samples (20–38%) compared to fat body samples (0.05 to 15%). Because of the abundance of 16SrRNA in the transcript data, it was removed from the dataset prior to downstream analysis. Global gene expression levels were assessed using RPKM values [43]. For further analyses, we filtered out genes in each set, muscles or fat body, which had less than an average of 10 reads per experiment. Approximately half of the 34,411 genes in the *de novo* transcriptome did not make this cutoff and thus were expressed at very low levels. This left 7909 genes in the muscle data set and 10441 in the fat body data set. Muscles and fat body expressed different sets of genes, with less than 33% overlap between the sets, suggesting that muscles and fat bodies are well- differentiated from one another at the gene expression level as expected from their divergent functions.

### Transcriptome data suggests major changes in metabolism between the pink and white flight muscles of the LW morph

The adult LW(f) morph of *G. firmus* has metabolically active and fully functional pink flight muscles [18]. These muscles are histolyzed to white flight muscles to produce the LW(h) morph starting on approximately the fifth day of adulthood. To identify the genes underlying physiological differences between these two muscle types, we compared the expression profiles of LW(f) pink and LW(h) white flight muscles. 3882 out of 7909 genes were significantly DE between the two muscle types using an FDR corrected *p*≤0.05 and fold change ≥1.5 cutoffs (Tables S5 and S6 and Figure S4). Of these 3882 genes, 373 showed higher expression in pink flight muscles and 3509 had higher expression in white flight muscles.

Many of the genes highly expressed in pink flight muscles belong to functional gene categories involved in cellular respiratory pathways. In fact, over-representation analysis of the 373 genes with higher expression in pink flight muscles yielded highly significant GO terms associated with biological processes such as oxidation-reduction, cellular respiration, and the electron transport chain, processes necessary for the greater metabolic requirements in these fully-functional muscles. The full list of over-represented GO terms and their significance values can be found in Table S7. An over-representation GO analysis of the 3509 genes with higher expression in white flight muscles yielded functional terms related to proteolysis as expected given that these white muscles are histolyzing (Table S8). The analysis also yielded other terms, such as those related to mitosis and the cell cycle. Thoracic muscles include flight muscles and also muscles used for activities such as walking. When flight muscles are histolyzed, the remaining thoracic muscles may need to compensate for their loss by making more muscle cells, which would in turn result in increased usage of genes involved in mitosis and the cell cycle.

Our results provide important molecular genetic insight into three previously found physiological and enzymatic differences between the LW(f) and LW(h) morphs. First, thoracic muscle respiration rate (i.e. oxygen uptake) is 39% higher in the LW(f) pink muscle relative to the LW(h) white muscle in *G. firmus* [18]. Our study identifies some of the specific genes that likely underlie these different respiration rates. Second, whole-muscle activities for enzymes involved in intermediary metabolism such as glycolytic and Krebs cycle enzymes are higher in LW(f) pink versus LW(h) white muscle, and this is especially the case for mitochondrial enzymes [44]. Our results show that these differences are apparent at the transcriptional level and are not the result of translational or post-translational differences between the morphs. And finally, mitochondria numbers are significantly lower in the SW morph (the LW(h) morph was not examined in this report [44]) relative to the LW(f) morph of *G. firmus*. Our gene expression profiling results are consistent with the idea that it is a decrease in the number of mitochondria that reduces thoracic muscle metabolism in the LW(h) morph relative to the LW(f) morph. Further precedent supporting such a scenario is provided by studies in muscle polymorphisms that exist in *Hydrophilus* and *Dytiscus* beetles where mitochondrial density is significantly reduced in the less active, white leg muscles when compared to flight musculature [45].

Our results also indicated that juvenile hormone diol kinase (Locus_141) was highly expressed in the white flight muscles when compared to the pink flight muscles. This gene encodes an enzyme that degrades juvenile hormone [46], suggesting that this hormone might be involved in morph-specific muscle histolysis.

LW(h) flight muscles are characterized by a 5–8 fold reduction in fiber area and a 40–50% reduction in fiber number [18]. Thus, one could reasonably expect to find higher expression of genes encoding proteins making up the sarcomere and/or associated structures (e.g. sarcoplasmatic reticulum, extra-cellular matrix, etc.) in the LW(f) when compared to LW(h) morph. However, we did not observe any enrichment for the category of genes encoding sarcomere proteins. This lack of enrichment may indicate that flight muscle histolysis in the LW(f) morph is a highly controlled process, with certain muscle fibers being selectively (and functionally) retained in the LW(h) morph flight muscles and little change to overall relative sarcomere gene expression (i.e. there is less muscle mass but it is maintained by the same relative gene expression pattern).

Even more surprising, we find that all but one transcript in a set of 20 sarcomere gene transcripts show higher expression in the LW(h) flight muscles. These transcripts, including key sarcomere genes such as myosin heavy chain, myosin light chain and troponin C, were likely insufficient in number to result in significant enrichment. This higher expression of sarcomere gene transcripts in LW(h) morphs could be because the histolysis of flight muscle is accompanied by a switch to different gene copies or alternative splice transcripts of the genes encoding myosin heavy chain and troponin C. Alternatively, the results could be due to changes in the alternative splicing patterns of these genes. Another interpretation is that the proteins encoded by these genes are more “sensitive” to the extensive proteolysis process that occur in LW(h) muscle and that our results are indicative of post-histolysis repair to the sarcomere to optimize remaining functionality.

In support of the first scenario, in *Drosophila* muscles myosin heavy chain is encoded by a single gene but is alternatively spliced to produce at least 20 isoforms [http://flybase.org/reports/FBgn0264695.html], while troponin C is encoded by (at least) four gene copies [47], [48]. Furthermore, our results indicate that four different transcripts annotated as troponin C exist in the *G. firmus de novo* transcriptome (Locus 26, 29, 1344 and 7475) and gene expression differences between the two morphs are seen in both directions. Loci 26, 29 and 1344 were highly expressed in LW(h) flight muscle, whereas Locus 7475 was highly expressed in LW(f) flight muscle (Tables S5 and S6). These four transcripts code for different protein sequences (but the nucleotide differences between theses four predicted transcripts have not yet been confirmed by Sanger sequencing) (Figure S5).

Thus, a troponin C gene expression shift appears to occur during the transition from LW(f) to LW(h), with a transition from higher expression for one of the transcripts in LW(f) functional, pink muscle to higher expression of the three others in LW(h) histolyzed, white muscle. Such a transition may be similar to the transition observed for troponin T in *L. pulchella* dragonflies, which have muscles that become more active as they mature. Flight muscles in this species show a maturational (during the teneral to adult transition) shift in troponin T protein isoform expression that affects flight muscle calcium sensitivity and performance, while expression of other sarcomere proteins remains stable [49].

### SW and LW(f) fat body exhibit transcriptional differences related to lipid biosynthesis and transport, immune function, and reproduction

Fat body is the major organ of intermediary metabolism in insects, and this organ differs between LW(f) and SW morphs in several important metabolic aspects related to the production of triglyceride flight fuel and eggs [3], [22], [28]. We compared the transcriptomes of fat body between LW(f) and SW morphs to identify the transcripts underlying differences in these aspects of metabolism, pooling the data from fat bodies of each morph collected in the morning (AM) and evening (PM) (an analysis of diel variation in transcript abundance will be considered elsewhere). Out of 10,441 transcripts in fat body, only 57 had significantly higher expression in LW(f) fat body and a similar number, 45, had significantly higher expression in SW fat body (Tables S9 and S10 and Figure S6). To rule out the possible effect of data pooling on the number of observed DE genes between the fat body of LW(f) and SW morphs, we also compared the fat body samples collected at the two different time points separately (LW(f)AM versus SWAM; LW(f)PM versus SWPM). These analyses also resulted in very few DE genes, with 93 and 100 from the AM and PM comparisons, respectively. Because of the small number of DE genes in fat body and sparse annotation information, we did not observe any significantly over-represented GO terms.

We independently verified the RNA-seq expression level data using qRT-PCR. We focused on four DE genes, annotated as an angiotensin converting enzyme, lectin related protein, an insulin related peptide precursor, and a glycerol-3phosphate acyl transferase, that had fold changes between the LW(f) and SW morphs ranging from 2.1 to 92.1. Fold changes between the two morphs determined by qRT-PCR were positively correlated with the RNA-Seq data (r^2^ = 0.99), suggesting that the RNA-Seq data are highly reliable (Table 3). Further, two of the genes that were not significant at the FDR-corrected p-value level of 0.05 were significant by qRT-PCR (Table 3), indicating the occurrence of false negatives in our results.

**Table 3:** Comparison of RNA-seq and qRT-PCR results of fat body transcripts.

Locus	Gene	RNAseq *p* value*	RNAseq *p* value, FDR corrected	Fold change	RT-PCR *p* value	Fold change
Locus_29388	Angiotensin converting enzyme	0.007	0.07	4.9× up in LW(f)	0.002	4.7× up in LW(f)
Locus_5558	Lectin related protein	7.41×10^−6^	0.00	92.1× down in LW(f)	0.0017	171.9× down in LW(f)
Locus_8270	Insulin related peptide precursor	0.0014	0.01	6.3× down in LW(f)	0.0001	6.8× down in LW(f)
Locus_2333	Glycerol-3phosphate acyl transferase	0.02	0.2	2.1× up in LW(f)	0.03	2.3× up in LW(f)

= FDR uncorrected t-test *p* value.

**Figure 2:**
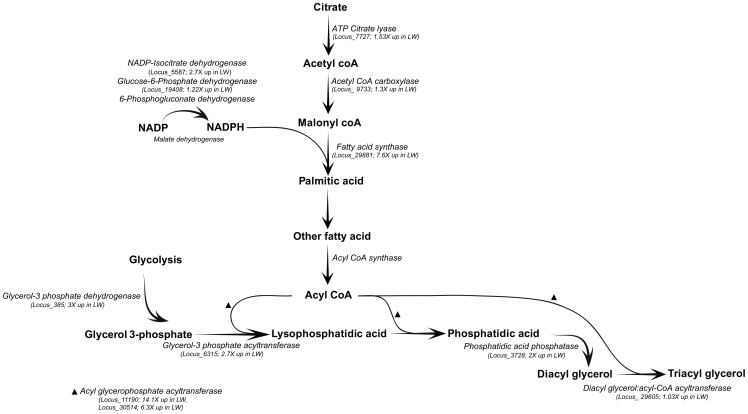
The major components of the fatty acid metabolism pathway. Fold changes of genes coding for the enzymes in this pathway in fat body of the SW and LW(f) morphs along with their locus ID are indicated. Expression of most of these genes was higher in the LW(f) when compared to SW morph.

Although LW(f) and SW fat body exhibited much fewer differences in transcript abundance than flight muscle, there were, nevertheless, a number of noteworthy differences. Three of the most important classes of transcript abundance differences and their associated genes are listed in Table 4. The first relates to differences in genes involved in lipid biosynthesis. Note that some of these genes have FDR-corrected p-values>0.05 (Table 4). We chose to include them because of our *a prior* interest in lipid biosynthesis and transport genes and because our qRT-PCR results indicated that some genes with FDR-corrected p-values>0.05 may be false negatives. Previous studies have demonstrated a substantially greater amount of total lipid and triglyceride in the LW(f) morph relative to the SW morph [21]. This is a key biochemical difference between the morphs: elevated lipid is required to fuel flight in the LW(f) morph, which, in turn, reduces allocation of nutrients to egg production, thus accounting for the reduced fecundity of the LW(f) morph. Elevated whole-body triglyceride is produced by elevated overall flux through the triglyceride biosynthetic pathway in the LW(f) morph [50]. This pathway is comprised of two components, the *de novo* fatty acid pathway, which produces fatty acids from the precursor acetyl CoA, and the glyceride pathway which links three fatty acids to a glycerol phosphate molecule. A number of transcripts involved in lipid biosynthesis and transport were elevated in LW(f) compared to SW females (Figure 2). For example, three enzymes of glyceride metabolism [glycerol-3-phosphate dehydrogenase, 1-acyl-3-glycerol-3-phosphate acyltransferase (two loci), and phosphatidate phosphatase, (two loci)], were elevated in LW(f) females. Several of these enzymes, such as phosphatidate phosphatase and 1-acyl-glycerol-3-phosphate acyltransferase, are membrane-bound proteins that are difficult to assay for activity [51], and activities have not previously been compared between the morphs. The present study has thus identified a key portion of the triglyceride biosynthetic pathway whose elevation likely accounts for the greater production of flight fuel in LW(f) females. Further, the availability of transcript sequences for these enzymes will be very useful for future studies of morph-specific triglyceride regulation, a major adaptive difference between the morphs [22], [23].

**Table 4:** Selected fat body transcripts that differ in abundance between LW(f) and SW morphs of *G. firmus*.

Transcript (Locus#)	Fold change
Lipid Biosynthesis and transport	
Glycerol-3-phosphate dehydrogenase (385)	3× up in LW!
1-acyl 3-glycerol-3-phosphate acyltransferase (2333)	2.2× up in LW!
1-acyl 3-glycerol-3-phosphate acyltransferase (111900)	14.1× up in LW***
Phosphatidate phosphatase (12931)	2.2× up in LW!
Phosphatidate phosphatase (3728)	2.3× up in LW!
Fatty acyl elongase (22361)	12.8× up in LW*
Fatty acyl reductase (10813)	9× up in LW!
Apolipoprotein-O-like (744)	1.8× up in LW!
Apolipophorin (5324)	2.5× up in LW*
Immune function	
Lectin-related protein (5558)	92.1× up in SW***
Lectin-related protein (30822)	13.4× up in SW**
Lectin-related protein (14597)	56.1× up in SW**
Lectin-related protein (31196)	21.6× up in SW*
Lectin-related protein (30749)	17.9×up in SW***
Trypsin inhibitor (14790)	5.2× up in SW***
Reproduction	
Insulin-related peptide precursor (8270)	6.3× up in SW*

FDR corrected ttest *p* values:

= *p*≤0.05,

= *p*<0.01,

= *p*<0.005.

^!^ = uncorrected ttest *p* value≤0.05.

Although we observed major differences in genes whose products are involved in the triglyceride biosynthetic pathway, we observed no significant difference in transcript abundances of enzymes of fatty acid biosynthesis (e.g., ATP-citrate lyase, fatty acid synthase; various NADP+dehydrogenases such as isocitrate, and 6-phosphogluconate dehydrogenases). These enzymes were uniformly, but modestly (1.7 fold) elevated in specific activity in LW(f) versus SW fat body in an earlier study [22], [24]. These low fold changes might be below the resolving power of the present study with only three biological replicates. Alternatively, the increase in specific activity of fatty acid biosynthetic enzymes in the LW(f) morph might be primarily due to post-transcriptional modifications.

The elevated abundance of fatty acyl elongase and reductase transcripts in LW(f) females indicates that fatty acids from the two morphs likely differ in both chain length and degree of saturation. The elevated transcript abundance of the lipid transport proteins, apolipoprotein-O and apolipophorin transcript, is consistent with the elevated locomotor capability of the flight-capable LW(f) morph (as mentioned, lipid is the main flight fuel in *Gryllus*). This lipid transporter may also function in lipid-fueled walking which occurs to a greater degree in LW versus SW morphs of some wing-polymorphic insects [52] including *G. firmus* (R.M. Clark, S. Behmer and A.J. Zera, unpublished).

Another important finding from the present study was the higher abundance of transcripts involved in immunity in the SW morph. Indeed, of all the annotated fat body transcripts in the present study, lectin-related protein transcripts differed the greatest in magnitude between the morphs, with lectin-related protein locus 5558 having 92.1× higher expression in the SW morph. Lectin comprises a family of related proteins that plays an important role in innate immunity in insects [53], [54]. In the desert locust, *Schistocerca gregaria*, the gregarious phase, which occurs in higher densities compared to the solitary phase, had a higher expression of immune related genes (including lectin-related gene) and is more resistant to fungal infections than the solitary morph [55], [56]. The SW morph of *G. firmus* also almost certainly occurs at higher densities in the field than the LW(f) morph (C. Mitra pers. comm.). Thus, the increased abundance of immune transcripts in the SW morph may function to combat increased probability of disease transmission due to crowding, as appears to be the case in the gregarious phase of locusts.

We also observed elevated abundance of transcripts of genes involved in reproduction and protein metabolism in SW fat body. Most noteworthy was the higher abundance of an insulin-like peptide in the SW morph. Insulin-like peptides have attracted considerable attention recently because they are phylogenetically conserved regulators of metabolism and reproduction, and are thought to play important roles in regulating life-history trade-offs [57], [58]. The insulin-related peptide precursor transcript variant t 1, which is in higher abundance in the more fecund SW morph of *G. firmus* (Table 4) is homologous to the transcript that is substantially elevated in the gregarious phase of the phase-polymorphic desert locust, *Schistocerca gregaria*, where it is correlated with earlier reproductive maturity of that morph [59]. The identification of this hormonal regulator in *G. firmus* opens the door to future investigations of morph-specific regulation of reproduction via insulin-like signaling peptides.

## Conclusion

Here we report the first *de novo* transcriptome for the wing polymorphic cricket species, *G. firmus*, an important model for understanding the mechanisms underlying life history adaptation. 34,411 transcripts were reconstructed from RNA taken from flight muscles and fat body, two organs that have been the foci of physiological and biochemical studies of morph adaptations in this species. We identified a large number of transcripts differentially expressed between functional and histolyzed flight muscles, with gene functions involved in cellular respiration and proteolysis. Genes associated with fatty acid metabolism, immunity and reproduction were differentially expressed between LW and SW fat body. Results from this study provide key information on the molecular mechanisms underlying physiological differences between morphs and will serve an invaluable resource for future investigations into the molecular mechanisms underlying morph adaptations in this and related species.

## Supporting Information

Figure S1 **Frequency distribution of the length range of the assembled transcripts.**
(EPS)

Figure S2 **Relationship between ortholog hit ratio (OHR) and assembly coverage (A, B) and** ***G. firmus*** **EST (C) and** ***G. bimaculatus*** **mRNA length (D).** Where this ratio is 1.0, the gene is likely fully assembled. Panels A and B show OHR's in terms of assembly coverage of the assembled transcripts, for comparison against the *G. firmus* EST library and the *G. bimaculatus* mRNA data set, respectively. The median OHR for the transcript matching EST's is 0.23, while the median OHR for those matching the G. bimaculatus mRNA's is 0.94. Panels C and D relate OHR to the length of the *G. firmus* EST's and *G. bimaculatus* mRNA, respectively. Overall, 55 out of the 291 transcripts that had a hit against the *G. firmus* EST's had an OHR>0.8, and 87 had an OHR>0.5. Of the 6542 transcripts with *G. bimaculatus* hits, 4271 had OHR>0.8, and 5421 had OHR>0.5.
(EPS)

Figure S3 **Species distribution of top BlastX results.** Blast2GO was used to Blast *G. firmus* transcripts against the non-redundant protein sequence database. Transcripts from *G. firmus* had high sequence similarity to *Tribolium castaneum*, *Pediculus humanus* and *Nasonia vitripennis*.
(EPS)

Figure S4 **Volcano plot of genes differentially expressed between pink and white flight muscles of the long-winged morph.** Darkened black dots represent genes having an FDR corrected *p* value≤0.05 and fold change ≥1.5.
(EPS)

Figure S5 **Comparison of multiple alignments and amino acid identity of putative troponin C transcripts in** ***G. firmus*** **(A) and** ***D. melanogaster*** **(B).** EF-hand loops (i.e. the calcium-binding regions) are marked with red lines (after Herranz et al., Gene Expr Patterns. 2004, 4(2):183–90.)
(EPS)

Figure S6 **Volcano plot of genes differentially expressed between the fat body of long-winged and short-winged morphs.** Darkened black dots represent genes having an FDR corrected *p* value≤0.05 and fold change ≥1.5. Red dots represent genes used for qRT-PCR.
(EPS)

Table S1 **Blast results, ortholog hit ratios, and coverage for each assembled transcript aligned against** ***G. firmus*** **EST's.** Note: Blastn e-value = 1e-10.
(XLSX)

Table S2 **Blast results, ortholog hit ratios, and coverage for each assembled transcript aligned against** ***G. bimaculatus*** **mRNA's.** Note: Blastn e-value = 1e-10.
(XLSX)

Table S3 **List of primer sequences designed for qRT-PCR.**
(DOC)

Table S4 **Assessing the completeness of the** ***de novo*** **transcriptome assembly.**
(DOCX)

Table S5 **Genes having significantly higher expression in pink flight muscles relative to white flight muscles** (FDR-corrected *p*≤0.05, fold change ≥1.5).
(XLS)

Table S6 **Genes having significantly higher expression in white flight muscles compared to pink flight muscles** (FDR-corrected *p*≤0.05, fold change ≥1.5).
(XLS)

Table S7 **Blast2GO enrichment analysis of genes highly expressed in pink flight muscles relative to white flight muscles.**
(XLS)

Table S8 **Blast2GO enrichment analysis of genes highly expressed in white flight muscles relative to pink flight muscles.**
(XLS)

Table S9 **Genes having significantly higher expression in LW fat body relative to SW fat body** (FDR-corrected *p*≤0.05, fold change ≥1.5).
(XLS)

Table S10 **Genes having significantly higher expression in SW fat body compared to LW fat body** (FDR-corrected *p*≤0.05, fold change ≥1.5).
(XLS)
