# Supplementary material for: De Novo Transcriptome Assembly from Fat Body and Flight Muscles Transcripts to Identify Morph-Specific Gene Expression Profiles in Gryllus firmus
Source: PLoS One. 2014 Jan 8;9(1):e82129. doi: 10.1371/journal.pone.0082129 (PMC3885399; doi:10.1371/journal.pone.0082129)
Supplement: Table S3 — List of primer sequences designed for qRT-PCR. (DOC) [file pone.0082129.s009.doc]

**Table S3**- List of primer sequences designed for qRT-PCR

| Locus | Gene | Forward primer 5' - 3' | Reverse primer 5' - 3' |
| --- | --- | --- | --- |
| Locus_29388 | Angiotensin converting enzyme | GCCGAGGACAAGAAGAACCT | GCCCAGAGCTCCTCGTAGT |
| Locus_5558 | Lectin related protein | CTGTGTGTCACCATCGCTCT | GTCCACAGAGGCCGAAATTA |
| Locus_8270 | Insulin related peptide precurser | TCCAAGTCCCAAAGAGAGGA | GGAAGGGATTTCAAGGGGTA |
| Locus_2333 | Glycerol-3phosphate acyl transferase | GTTGTCACTGGGCTTTGGTT | TTATTGAAGCATTGCCACCA |
| Locus_2182 | Vesicle trafficking protein (control) | AACACCCCAAAGTCGAAGTG | TCCATGTTGTGGAACTGCAT |
| Locus_5663 | Calcium binding protein (control) | AATTCGGTCTCCGAGAGGAT | CGCCCAAATTCAAGAAGAAA |
| Locus_938 | Actin (control) | TTCAGGCTGTCCTGTCACTG | ATTTCTCGCTCAGCAGTGGT |
|  |  |  |  |
