# Supplementary material for: De Novo Transcriptome Assembly from Fat Body and Flight Muscles Transcripts to Identify Morph-Specific Gene Expression Profiles in Gryllus firmus
Source: PLoS One. 2014 Jan 8;9(1):e82129. doi: 10.1371/journal.pone.0082129 (PMC3885399; doi:10.1371/journal.pone.0082129)
Supplement: Table S4 — Assessing the completeness of the de novo transcriptome assembly. (DOCX) [file pone.0082129.s010.docx]

**Table S4 – Assessing the completeness of the *de novo* transcriptome assembly**

| Percent of reads used | mRNA loci detected | Transcripts found |
| --- | --- | --- |
| 10  20  30  40  50  60  70  80  90  100 | 5722  11097  15590  19451  22599  25666  28285  30360  32236  34411 | 6152  11715  16524  21010  24081  27402  30575  32867  34824  37391 |

To determine to completeness, with regards of total number of genes found, de novo assemblies were performed with different percentages of randomly selected reads. The Oases reported mRNA loci and transcripts indicate that there is still a significant increase in the number of genes detected.
